# Longterm endocrine effects of cancer

**DOI:** 10.1186/1687-9856-2013-S1-O18

**Published:** 2013-10-03

**Authors:** Margaret Zacharin

**Affiliations:** 1 Dept of Endocrinology, Royal Children’s Hospital, Parkville , Victoria, Australia

## 

Survival after childhood cancer has increased over the last 25 years with around 80% overall survival but outlook is not trouble free. Within 25 years of diagnosis, 4% will have a second tumour. Cardiotoxicity from anthracyclines, radiation related health problems and endocrine deficiencies all contribute to increased mortality, with 18% reported to have died 30 years after diagnosis. Memory processing deficits after childhood cranial radiation result in additional burdens of intellectual, psychosocial and emotional disability.

Endocrine late effects of radiation and chemotherapy can be direct, resulting in hypofunction of endocrine glands or indirect resulting in metaplasia, cancer and altered bone growth. Deficiencies of hypothalamic pituitary hormones can be expected in 60-100% of patients by 8-10 years after radiation exposure.

Pubertal timing and tempo is altered by cranial radiation, with combinations of early puberty and growth hormone deficiency causing diagnostic and management confusion. Evolution of hormonal losses need be recognized.

Gonadal dysfunction occurs after both radiation and chemotherapy at any age.The prepubertal testis is not protected from effects of chemotherapy. Age at treatment, type and dose all predict outcome. Loss of function in germinal epithelium and Leydig cells is not reflected in gonadotrophin alterations until age 9-10 years. Abnormal timing of menarche in survivors of central nervous system tumours is common. Follicular reserve in young females after cancer treatment is reduced with high risk for premature menopause and little evidence that the ovary can be protected from insult, although 50% of children exposed to early radiation or chemotherapy retain sufficient gonadal function to initiate or complete puberty. Preservation of gonadal tissue prior to cancer treatment is now a priority, with semen storage, or gonadal biopsy being offered or considered in both sexes before exposure to gonadotoxins.

However, fertility recovery can occur and appropriate contraceptive needs must be met. Cerebral arteritis occurs after brain radiation, with early cerebrovascular accident. HRT may need to be tailored to reduce this risk.

Solid organ radiation exposure is hazardous. Thyroid cancer after radiation exposure occurs at 20 times population risk, with regular thyroid ultrasound every 2 years now recommended and FNA as required. Pelvic radiation results in poor pregnancy outcomes with increased foetal loss and small for dates infants. Bladder risks for cancer after cyclophosphamide, abdominal and pelvic radiation are worsened by smoking and alcohol. Bowel risks are currently unknown.

1 in 700 adults is now a survivor of childhood cancer and 60% have at least one chronic health problem. Ongoing surveillance is required to improve outcomes for this group of young people.
